# Structural analysis of previously unknown natural products using computational methods

**DOI:** 10.1007/s11418-022-01637-y

**Published:** 2022-07-18

**Authors:** Hikaru Kato

**Affiliations:** Graduate School of Pharmaceutical Sciences, Kumamoto University, Oe-honmachi 5-1, Kumamoto, 862-0973 Japan

**Keywords:** Natural products, Structure analysis, Computational chemistry, Absolute configuration, Electronic circular dichroism spectrum, Stereoisomer

## Abstract

Natural products exhibit structural diversity, and biologically active natural products with unprecedented molecular skeletons can potentially be isolated from natural resources in the future. Although it has often been difficult to determine the structures and configurations of new compounds that do not resemble known compounds, the determination of the chemical structures, including the absolute stereo configuration, is very important in drug discovery research. In our efforts to find new bioactive natural products, we have identified novel compounds such as the ubiquitin–proteasome system inhibitors and osteoclast differentiation inhibitors. Various natural products, mixtures of stereoisomers of natural products, and compounds with novel skeletal structures were studied. In cases where it was difficult to determine the structures by NMR spectroscopy, we could successfully determine the chemical structures by computational chemistry. This review presents the results of structural analysis obtained using computational methods for several natural products that we have recently isolated.

## Introduction

Natural products exhibit structural diversity and have been widely used for the development of pharmaceuticals. Detailed knowledge is essential for effective determination of the planar structure, relative configuration, and absolute configuration of potentially useful, naturally occurring compounds. The structural analyses of bioactive natural products that are structurally similar to known compounds are easier than that of compounds that do not resemble known compounds. It is also difficult to determine the absolute configuration of natural products if they consist of a mixture of stereoisomers.

We have previously identified bioactive natural products such as the ubiquitin–proteasome system inhibitors [1–16] and osteoclast differentiation inhibitors [17–22]. Some of those natural products were compounds with unique substructures or mixtures of stereoisomers such as epimers or diastereomers. It was difficult to determine the structures and configurations of these complex compounds by NMR spectroscopy, because not enough information was available. However, we could successfully determine the chemical structures by computational chemistry. In this review, we describe techniques of structure determination for previously unknown natural products using computational methods.

## Taichunamide C (1): diketopiperazine isolated from the fungus *Aspergillus taichungensis*

**Fig. 1:**
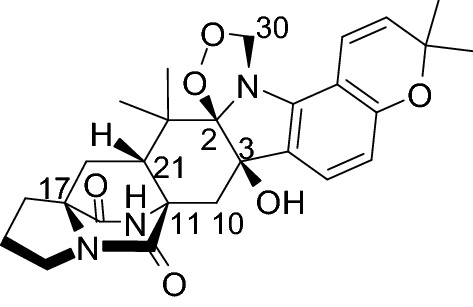
Structure of compound **1**

**Fig. 2:**
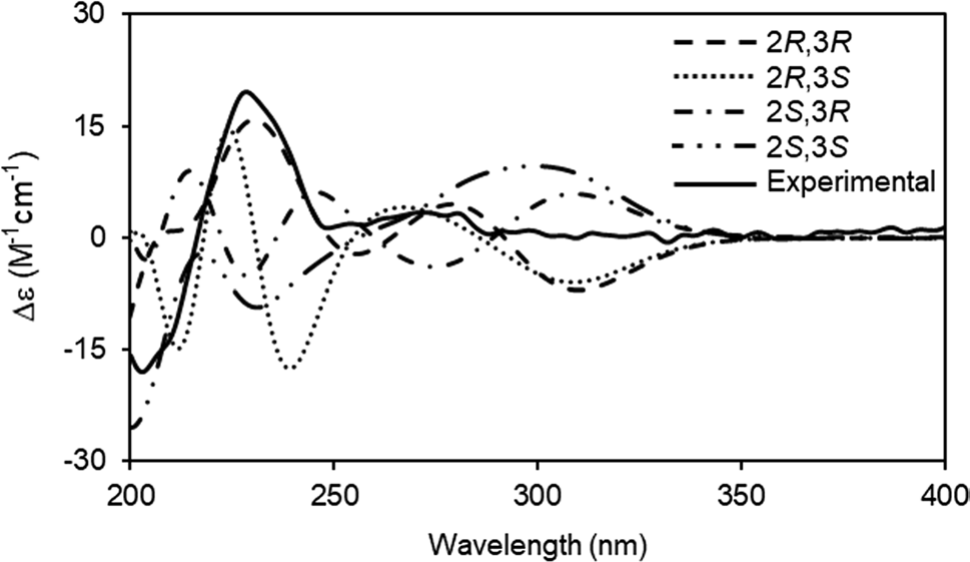
Experimentally obtained electronic circular dichroism (ECD) profile of **1** and calculated spectral profile of the four stereoisomers

Notoamide analogs are isolated from *Aspergillus* fungi, and they contain tryptophan–proline diketopiperazine. We have isolated approximately 50 new notoamide analogs to date [23–32], and the biosynthetic mechanism of these alkaloids has also been studied [25–29, 32–34]. Seven structurally novel analogs, isolated from the metabolites of *A*. *taichungensis* (IBT19404), were analyzed, and their structures were determined [30]. Among these seven new analogs, compound **1** was the first natural product with a 1,2,4-dioxazolidine ring (Fig. 1). The absolute configuration of the bicyclic ring could be determined by analyzing the electronic circular dichroism (ECD) profiles recorded for the compound, because many known analogs have the same partial structure. Significant nuclear NOE correlation, such as between H-10 and H-30, was not observed during the determination of the relative configuration between C-2 and C-3. The relative configuration could not be determined by analyzing the NMR spectra of the compounds, because we were the first to report a compound containing a 1,2,4-dioxazolidine ring in the core structure, and reference spectra were not available in the literature. We decided to use computational methods to determine the absolute configuration. Since the absolute configurations of the centers, except for the C-2 and C-3 centers, were clear as described above, the possibility of the presence of four types of stereoisomers (2*R*,3*R*-, 2*R*,3*S*-, 2*S*,3*R*-, and 2*S*,3*S*-**1**) was investigated. Molecular mechanics conformational searches were performed for the four isomers using Merck molecular force field (MMFF), then density functional theory (DFT)-based calculations were used to optimize the structures of the obtained conformations. The calculated ECD profiles of the four stereoisomers were obtained using the time-dependent density functional theory (TDDFT) technique at the B3LYP/6-31G* level. The characteristic positive Cotton effect observed at 230 nm in the experimentally obtained ECD profile recorded for **1** agreed well with the effect observed in the calculated ECD profiles recorded for 2*R*,3*R*-**1** (Fig. 2). Therefore, it could be concluded that the absolute configuration of **1** was 2*R*,3*R*,11*S*,17*S*,21*R*. The same conclusions could be drawn from the results obtained by calculations performed at the CAM-B3LYP and BHandHLYP levels [30].

## Sulawesin A (2): a mixture of four diastereomers isolated from a marine sponge (*Psammocinia* sp.)

**Fig. 3:**
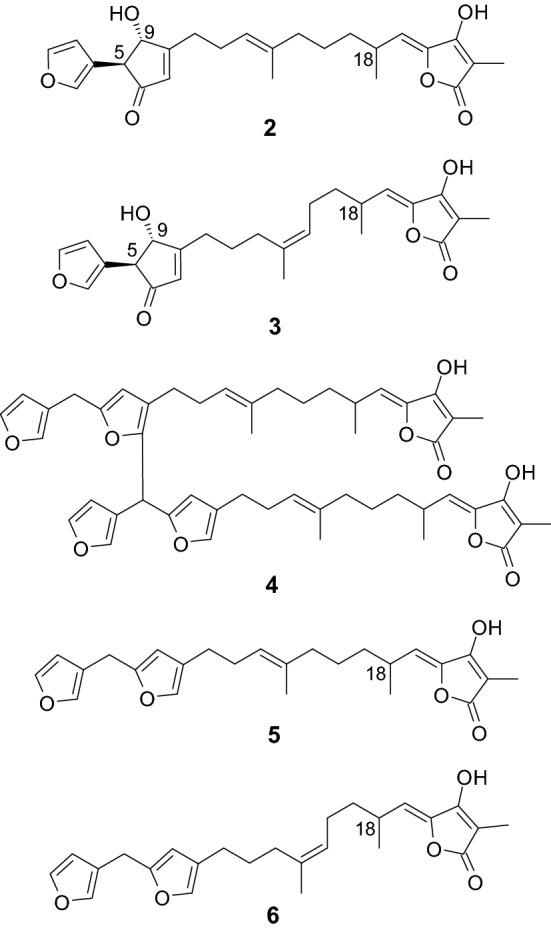
Structures of **2**–**6**

We isolated three new furanosesterterpene tetronic acids (sulawesins A–C (**2**–**4**)) and two known analogs (ircinins 1 and 2 (**5** and **6**)) (Fig. 3) from the *Psammocinia* sponge (North Sulawesi, Indonesia, September 2007) [12]. A comparison of the specific rotation values ([*α*]_D_) of compounds **5** and **6** (+ 8.0 (*c* 0.050, MeOH) and − 24 (*c* 0.050, MeOH), respectively) obtained by us with the values presented in the literature ((+)-**5**, [*α*]_D_ =  + 32.3 (c 0.050, MeOH); (+)-**6**, [*α*]_D_ = + 34.8 (*c* 0.060, MeOH) [35]; (−)-18*S*-**5**, [*α*]_D_ = − 34.12 (MeOH); (−)-18*S*-**6**, [*α*]_D_ = − 40.20 (MeOH) [36]) indicated the presence of an optical mixture. We proved this hypothesis by isolating the (+)- and (−)-forms of the compound using a chiral column. This was the first time that **5** and **6** were obtained as optical mixtures (mixture of epimers; epimerization at C-18). It was presumed that compounds **2**–**4** consisted of epimers (epimerization at C-18).

**Fig. 4:**
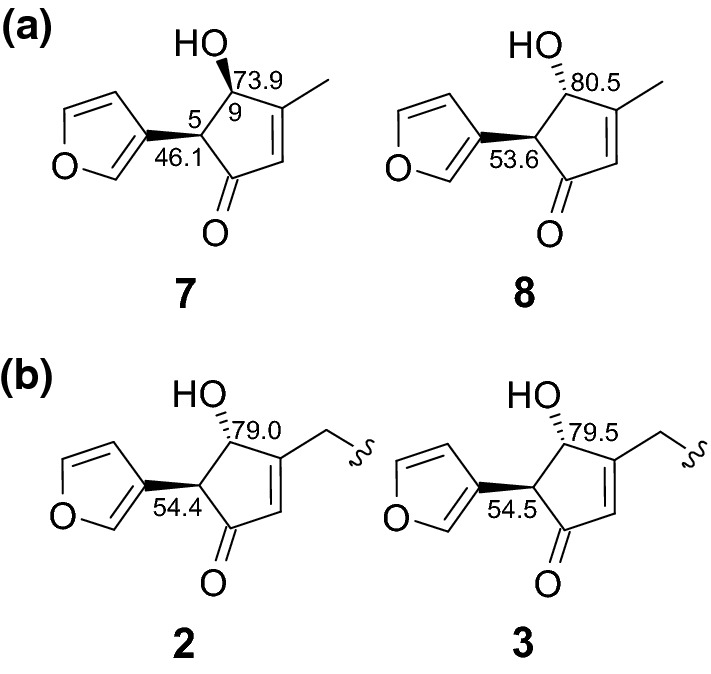
**a** Calculated ^13^C NMR chemical shifts for **7** and **8** (EDF2/6-31G*). **b** Experimentally obtained ^13^C NMR chemical shifts for **2** and **3**

**Fig. 5:**
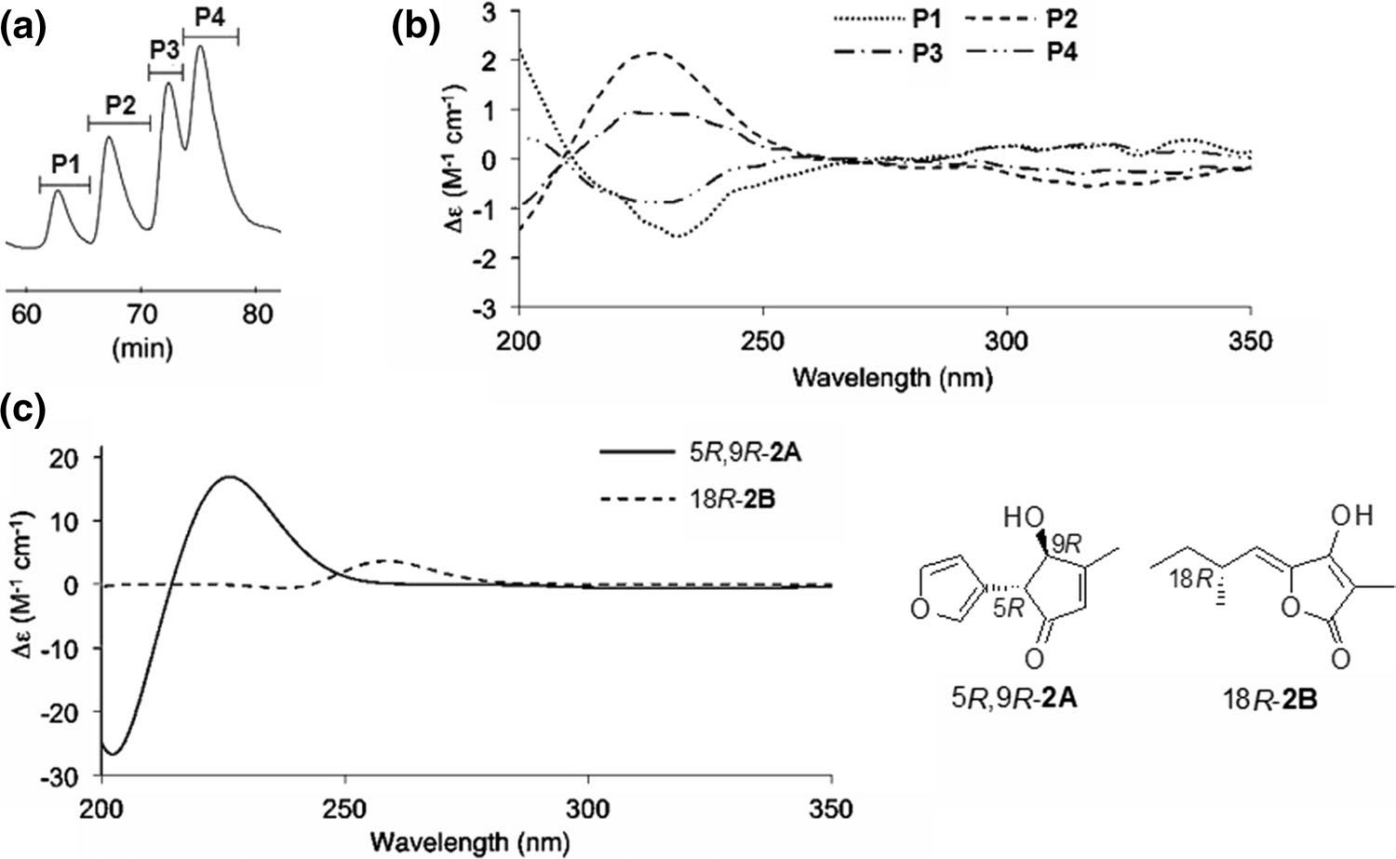
**a** Chiral HPLC chromatograms recorded for **2**. **b** Experimentally obtained ECD profiles of P1–P4. **c** Calculated ECD profiles of 5*R*,9*R*-**2A** and 18*R*-**2B** (B3LYP/6-31G*)

The planar structures of **2** and **3** were determined by analyzing various spectral profiles. The ^13^C NMR chemical shifts of the structurally simple model compounds **7** (*cis*) and **8** (*trans*) (Fig. 4a) were calculated, and the chemical shifts were analyzed to determine the relative configurations at C-5 and C-9. The calculated chemical shifts corresponding to the C-5 and C-9 centers of the *cis*-isomer were significantly different from the calculated chemical shifts corresponding to the C-5 and C-9 centers of the *trans*-isomer. The experimentally obtained chemical shifts corresponding to the C-5 and C-9 centers in **2** and **3** matched well with the values calculated for the centers in the *trans*-isomer. This suggested that C-5/C-9 (in **2** and **3**) was *trans* (Fig. 4b). Since it was presumed that **2** and **3** consisted of a mixture of epimers, the compounds were analyzed by HPLC with a chiral column. Four peaks (P1–P4) were detected in the chromatogram recorded for **2** (Fig. 5a), and the same result was obtained for **3**. This suggested that each compound was a mixture of four diastereomers (5*R*,9*R*,18*R*, 5*R*,9*R*,18*S*, 5*S*,9*S*,18*R*, and 5*S*,9*S*,18*S*). An analysis of the ECD profiles of P1–P4 revealed the characteristic Cotton effect near 225 nm (Fig. 5b). Since **2** and **3** contained two chromophore units each, we investigated which chromophore corresponded to the peak at 225 nm. We designed 5*R*,9*R*-**2A** and 18*R*-**2B** as simplified model compounds and theoretically generated the ECD profiles for each compound. 5*R*,9*R*-**2A** exhibited a strong Cotton effect at 225 nm. The absolute configuration could be determined by analyzing the peak at 225 nm. The ECD profiles showed positive (for **2b**/**2c**) and negative (for **2a**/**2d**) Cotton effects at 225 nm, which suggested that the configuration of **2b**/**2c** was 5*R*,9R and that of **2a**/**2d** was 5*S*,9*S*. The absolute configuration at C-18 could not be determined, because the peak at 260 nm in the profile recorded for **2B** was low in intensity. It was considered that the peak was hardly observed in the experimental ECD spectrum (Fig. 5b and c). Although **2** contained four diastereomers, the presence of the diastereomers was not observed by the NMR spectrum. Furthermore, analysis of the ECD profiles did not reveal the interaction between the two chromophores present in **2**. These observations indicated that the two chromophores were connected by a long carbon chain. Hence, they did not influence each other.

## Niphateolide A (9): a diterpene isolated from the marine sponge *Niphates olemda*

**Fig. 6:**
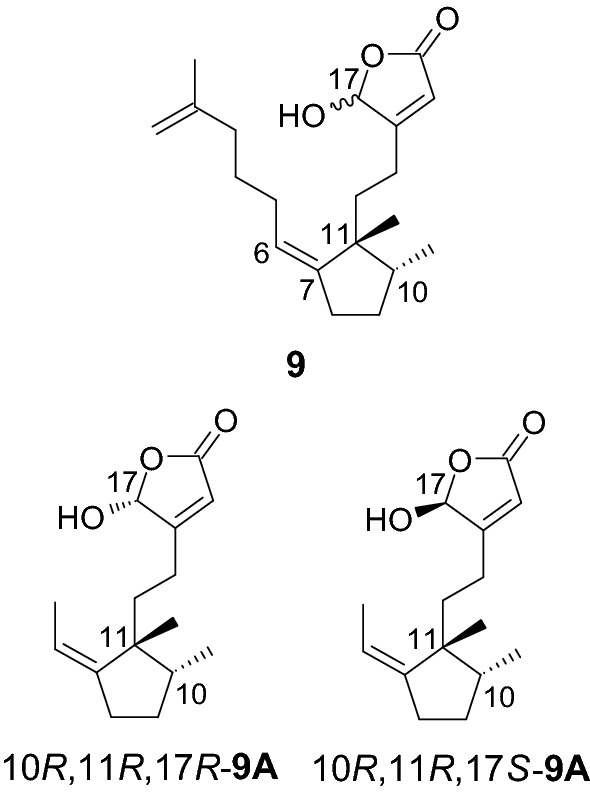
Structures of **9**, 10*R*,11*R*,17*R*-, and 10*R*,11*R*,17*S*-**9A**

**Fig. 7:**
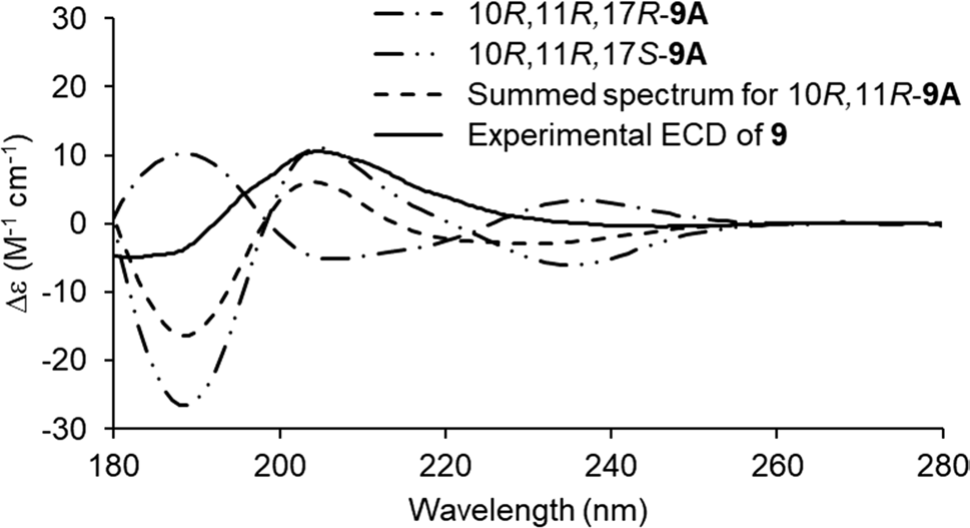
Calculated ECD profiles of 10*R*,11*R*,17*R*-**9A** and 10*R*,11*R*,17*S*-**9A** (B3LYP/6-31G*), summed spectrum for 10*R*,11*R*-**9A** calculated based on their abundance ratio, and experimentally obtained VUV-ECD profile of **9**

We isolated a new compound, niphateolide A (**9**) (Fig. 6), from the ethanol extract of the sponge *Niphates olemda* (Mantehage, North Sulawesi, Indonesia, December 2006) [10]. The analysis of various NMR spectra and molecular weights suggested that **9** was a diterpene containing a cyclopentylidene moiety and a *γ*-hydroxybutenolide moiety. It was a mixture of C-17 epimers (1:1). The NOE effect was analyzed to reveal that the configuration of **9** was 10R*,11R*. The ECD spectrum was generated computationally to determine the absolute configuration. Particular attention was paid to the following points. (1) The ECD profiles were usually recorded at wavelengths longer than 200 nm. Exciton-split CD profiles resulting from the correlation between the exocyclic double bond (C-6/C-7; 185 nm) and *γ*-hydroxybutenolide (207 nm) [37] were observed partially in the range beyond 200 nm. Therefore, we recorded the vacuum ultraviolet (VUV)-ECD spectrum at wavelengths shorter than 200 nm. (2) Since it was concluded that **9** was a mixture of epimers (epimerization at C-17), we theoretically generated the ECD profiles (at the B3LYP/6-31G* level) of the two epimers (10*R*,11*R*,17*R*- and 10*R*,11*R*,17*S*-**9A**). The spectral profile obtained by adding the two calculated ECD profiles (for 10*R*,11*R*-**9A**) reproduced the experimentally obtained spectral profile of **9** (Fig. 7). Hence, the absolute configuration of **9** could be determined as 10*R*,11*R*. The same conclusions were reached when different functionals, such as CAM-B3LYP and BHandHLP, were used for the calculations. For a mixture of stereoisomers, the experimentally obtained ECD profile could be reproduced by adding the calculated ECD profiles of each isomer.

## Conclusion

In this study, we used computational methods to elucidate the chemical structures of natural products with partial structures that were previously unknown. The structures of natural products containing a mixture of stereoisomers, whose structures were difficult to determine solely by NMR spectroscopy, were also determined.

It is difficult to analyze the structure of new compounds with unprecedented molecular skeletons, because reference data are not available. In such cases (e.g., taichunamide C (**1**)), computational methods can be used to generate the ^13^C NMR spectra and ECD profiles. The computationally obtained spectra can be compared with the experimentally obtained spectra to determine the structure of the compounds. We observed that the ECD profiles of the sulawesins (**2**–**4**) and niphateolide A (**9**) were the summations of spectra of the Cotton effects observed for each chromophore. We also succeeded in determining the configuration when the compound consisted of a mixture of stereoisomers.

NMR spectroscopy and X-ray crystallography are commonly used to determine the structural properties of natural products. However, rapid and accurate determination of structural features may be achieved using computational methods, which may be particularly useful for analyzing previously unknown compounds. Natural products that can be used as safer and more effective pharmaceuticals may be identified using advanced structural analysis and computational techniques.
